# Thyroxine Regulates the Opening of the Organ of Corti through Affecting P-Cadherin and Acetylated Microtubule

**DOI:** 10.3390/ijms232113339

**Published:** 2022-11-01

**Authors:** Huimin Zhang, Le Xie, Sen Chen, Yue Qiu, Yu Sun, Weijia Kong

**Affiliations:** 1 Department of Otorhinolaryngology, Union Hospital, Tongji Medical College, Huazhong University of Science and Technology, Wuhan 430022, China; 2 Institute of Otorhinolaryngology, Tongji Medical College, Huazhong University of Science and Technology, Wuhan 430022, China

**Keywords:** pillar cell, development, T3, microtubule acetylation, P-cadherin

## Abstract

Different serum thyroxine levels may influence the morphology of the inner ear during development. A well-developed organ of Corti (OC) is considered to be critical to the function of hearing. In our study, we treated mice with triiodothyronine (T3) and found that the opening of the OC occurred sooner than in control mice. We also observed an increased formation of acetylated microtubules and a decrease in the adhesion junction molecule P-cadherin the during opening of the OC. Our investigation indicates that thyroxin affects P-cadherin expression and microtubule acetylation to influence the opening of the OC.

## 1. Introduction

Thyroxine is important for the development of hearing. Either abnormal serum thyroxine levels, i.e., hyperthyroidism, hypothyroidism or a deficiency in thyroid hormone transporters, such as Slc16a2 and Slc16a10, may cause cochlea development disorders and hearing loss [1,2]. In addition, thyroid-hormone-activating enzymes, such as type 2 deiodinase (Dio2) deficiency, may also cause deafness and hypothyroid-like cochlear defects [3]. The latest research reported Dio2 expression in cochlear fibrocytes and osteoblasts, which revealed how the thyroid hormone in the circulation reaches internal sensory tissues in the cochlea [4]. Within two weeks after birth in mice, the cochlear structure is progressively formed, and the hearing function is basically developed. Interestingly, in mice administered thyroxine, the inner ear structure develops earlier, and the organ of Corti (OC) opens earlier in the inner ear [5]. Thyroxine is capable of affecting the opening of the OC during the early stage of cochlear development, and a low thyroxine level delays the development of the cochlear structure and triggers hearing loss [6]. These findings indicate that thyroxine might mediate the opening of the OC by regulating the pillar cells (PCs), but this mechanism is poorly understood.

The development of inner and outer PCs is reported to be a vital marker of the maturation of the OC. The fibroblast growth factor (FGF) signal had been reported to play critical roles in PC differentiation [7]. PCs gradually separate to form pillar-like cell bodies. The morphological variations of supporting cells determine the normal function exhibited by the cochlea [8,9]. In the OC, there are a wide range of molecular structures of the homotypic or heterotypic cell junctions (e.g., tight junction, adhesion junction, desmosomes and gap junction) [10], and the cleavage of cadherin plays an important role in the opening of the OC [11]. Meanwhile, acetylation is the main post-translational modification of tubulin, and occurs in a unique time-dependent manner in PC development, while acetylated tubulin levels are increased in adult mouse cochlea, especially in supporting cells [12]. Although these studies revealed that the cytoskeletal system (e.g., acetylated tubulin and adhesion molecules) jointly affects the maturation of PCs and then participates in the normal opening of the OC, the specific molecular interaction mechanism of this process remains unclear.

To explore the regulatory mechanism of PCs and its correlation with thyroxine, we used normal mice and thyroxine-induced mice to examine changes in inner ear development and the role of cytoskeletal molecules and adhesion molecules in the opening of the OC.

## 2. Results

### 2.1. Normal Opening of the TC and Early Opening Promoted by T3

**Figure 1:**
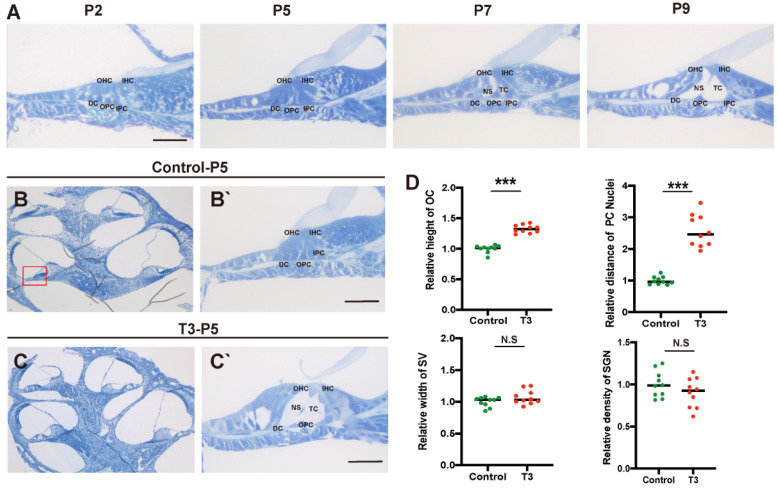
Normal opening of the TC and early opening promoted by T3. (**A**) The OC structure of the normal inner ear at P2, P5, P7 and P9. Plane (**B**,**C**) shows full views of the cochleae and the middle turn in the organ of Corti; (red frame, (**B’**,**C’**)) magnified images for detailed investigation, showing a closed TC from the P5 control group and an opened TC from the P5 T3 group. (**D**) Relative heights of TCs in the control and T3 groups (*n =* 3). Relative distance between the nuclei of IPCs and OPCs in the control and T3 groups (*n =* 3). Relative SV width of TCs in the control and T3 groups (*n =* 3). Relative SGN number in the control and T3 groups (*n =* 3). Significantly different from the control group (*** *p* < 0.001). N.S means no significance. OC: organ of Corti, TC: tunnel of Corti, NS: Nuel’s space, OPC: outer pillar cell, IPC: inner pillar cell, OHC: outer hair cell, IHC: inner hair cell, DC: Deiters’ cell, SV: stria vascularis, SGN: spiral ganglion neuron. The scales in panel (**A**,**B’**,**C’**) represent 40 μm.

Toluidine blue staining was used to observe the normal development of the mouse cochlea (postnatal day (P)2, P5, P7, and P9; Figure 1A). According to the results, except for the increase in the number of stria vascularis and spiral ganglion cells, there were significant morphological variations in the OC after birth, and the tunnel of Corti (TC), Nuel’s space and outer tunnel in P7 mice opened obviously. The TC, Nuel’s space and outer tunnel tended to open with age. The cochlea of the T3-treated and control mice was also identified (Figure 1B,C). At P5, the TC, Nuel’s space and outer tunnel of the cochlea, from the parietal to the basal circle in the T3-treated mice, opened completely. Compared with the controls, the height of the TC in the T3 group increased by 32.6 ± 0.03% (*n* = 3 in each group, *p* < 0.0001). The distance between the nuclei of the inner pillar cells (IPCs) and the outer pillar cells (OPCs) increased by 157.3 ± 16.6%, compared with the controls (*n* = 3 in each group, *p* = 0.0003). Furthermore, the opening area of the TC increased (Figure 1D). All structures (Figure 1C) formed a cochlea morphology consistent with that of the normally developing mice at P11, while the spaces of the cochlea of the control mice were not fully open at P5.

### 2.2. The Ultrastructure of PCs Changes Significantly after T3 Administration

**Figure 2:**
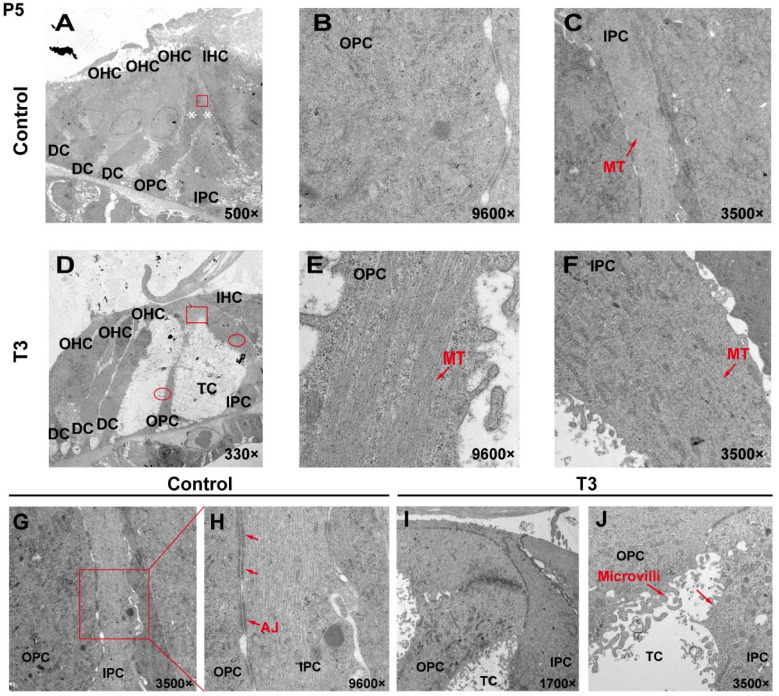
The ultrastructure of PCs changes significantly after T3 administration. (**A**,**D**) Transmission electron micrograph showing a closed TC from the P5 control group and an opened TC from the P5 T3 group (White asterisk indicate the location of PCs of (**B**,**C**)). Plane (**B**,**C**) transmission electron micrograph showing OPCs and IPCs of control mice, and Plane (**E**,**F**) showing the OPCs and IPCs of T3-induced mice. Plane (**G**,**H**) the junction of OPCs and IPCs from control mice, and Plane (**I**,**J**), the junction of OPCs and IPCs from T3-induced mice. MT: microtubule; AJ: adherens junction.

To analyze the effect of T3 on the ultrastructure of the OC, the cell ultrastructure of the cross-sections of the OC was examined in the cochlea of the control and the T3 group by transmission electron microscopy (Figure 2A,D). At P5, the OC in the group treated with T3 was larger than that of the normal OC. The supporting cells forming the TC structure, i.e., the IPCs and OPCs, were also observed. In the T3 group, the structures of adherens junctions connecting the IPCs and OPCs were not visible (Figure 2I,J). In contrast, the TC in the control was closed, and significant adhesion and connection were reported between the IPCs and OPCs (red rectangle in Figure 2G, enlarged in Figure 2H).

In the T3 group, there were considerable bundles of microtubules in the cell body of the OPCs (red arrow, Figure 2E), while there were fewer bundles of microtubules in the control group (Figure 2B). However, the microtubules in the IPCs were identical between the two groups (Figure 2C,F). It is noteworthy that the morphology of the microtubules in the OPCs of the T3 group was more diversified than that of the treatment group, the arrangement was worse than that of the T3 group and the cell shape was not as straight as that of the T3 group (Figure 2E).

### 2.3. T3 Promotes the Separation of IPCs and OPCs by Affecting P-Cadherin Transfer into the Cytoplasm from the Cell Membrane

**Figure 3:**
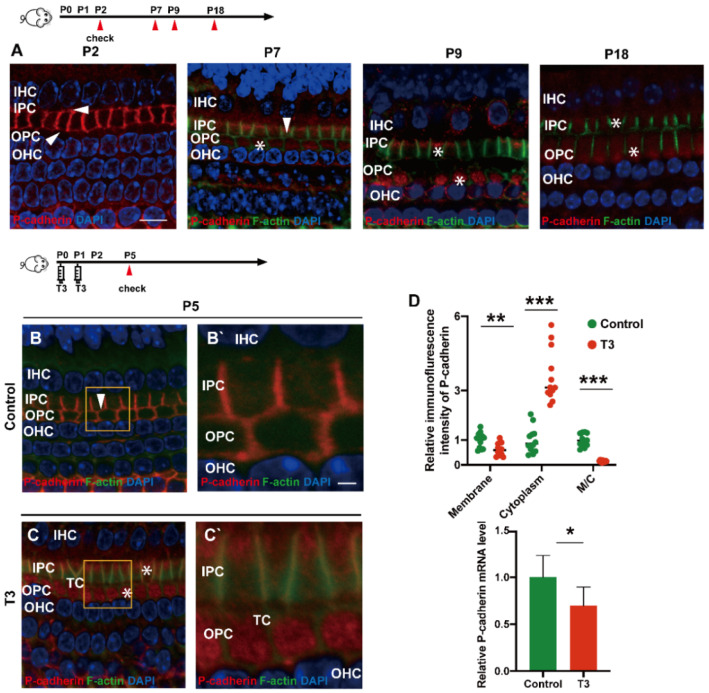
T3 promotes the separation of IPCs and OPCs by affecting P-cadherin transfer into the cytoplasm from the cell membrane. (**A**) Staining of F-actin (green) and P-cadherin (red) in hair cells of the P2, P5, P7 and P9 groups, respectively. Nuclei were counterstained with DAPI (4′,6-diamidino-2-phenylindole, blue). (**B**,**C**) F-actin (green) and P-cadherin (red) in the hair cell nuclei (blue) of the P5 control and P5 T3 group, respectively. (**B’**,**C’**) Magnified areas for detailed investigation from (**B**,**C**). (**D**) Relative P-cadherin mRNA level of the cochlear basilar membrane in the control and T3 groups (*n =* 6 in each group, * *p* < 0.05). Quantification of P-cadherin fluorescence in the PC membrane (M), cytoplasm (E) and M/C at the level of the hair cell nuclei (*n =* 3 in each group, ** *p* < 0.01, *** *p* < 0.001). White asterisk and white arrow indicate expression of P-cadherin in the cell membrane and cytoplasm, respectively. The scales in panel (**A**) and (**B’**) represent 10 μm and 2 μm, respectively.

During the development of the OC, P (placental)-cadherin was expressed in different parts of the IPCs and OPCs. Before the opening of the TC (P5), P-cadherin was largely expressed on the surface of the cell membrane of the IPCs and OPCs, Deiters’ cells and Claudius cells. However, P-cadherin was transferred from the cell membrane to the cytoplasm of the PCs around P7. Expression of P-cadherin was also higher in the cytoplasm than in the cell membrane. Notably, P-cadherin was not co-located with phalloidin between the IPCs and OPCs, or between the OPCs and OPCs, i.e., after the TC opened, P-cadherin was expressed in the cytoplasmic region below the membrane of filamentous actin (F-actin). After the TC was opened, P-cadherin remained in the cytoplasm for a certain period (Figure 3A). Subsequently, some P-cadherin was present on the cell membrane of the adjacent IPCs.

T3 promoted the earlier opening of the TC. Compared with the control, the immunofluorescence-labeled P-cadherin on the membrane surface of the OPCs in the T3 group decreased significantly by 36.93 ± 11.75% (*p* = 0.0047). In contrast, the immunofluorescence of P-cadherin in the cytoplasm of the PCs increased significantly by 258.1 ± 34.39% (*p* < 0.0001, Figure 3D). The analysis also indicated that the expression ratio of P-cadherin on the membrane of the OPCs to that in the cytoplasm of the PCs significantly decreased by 84.38 ± 7.44% (Figure 3D; *p* < 0.0001). Moreover, mRNA was extracted from the basilar membrane of the T3 group and the control, and a relative decrease by 30.34 ± 12.45% of P-cadherin mRNA expression in the basilar membrane after T3 treatment was verified by real-time quantitative polymerase chain reaction (PCR) (Figure 3D; *p* = 0.0351).

### 2.4. E-Cadherin and N-Cadherin Do Not Change Significantly during Opening of the TC

**Figure 4:**
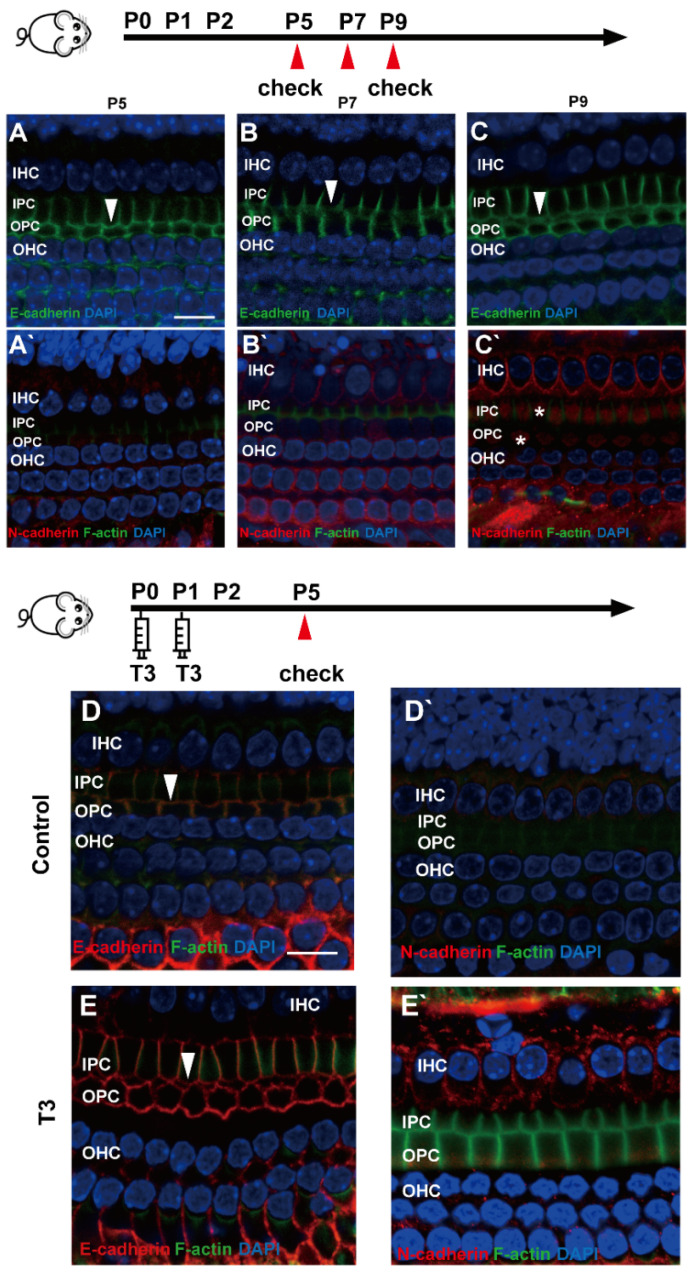
E-cadherin and N-cadherin do not change significantly during opening of the TC. (**A**–**C**) Staining of E-cadherin (green) in the hair cells of the P5, P7 and P9 groups, respectively. Nuclei were counterstained with DAPI (blue). (**A**’–**C**’) Staining of N-cadherin (red) in the hair cell nuclei (blue) in the P5, P7 and P9 groups (White asterisk showed N-cadherin expressed in pillar cells cytoplasm, white arrow showed E-cadherin expressed on pillar cells membrane). (**D**,**E**) Staining of E-cadherin (red) in the hair cell nuclei (blue) of the P5 control and P5 T3 group, respectively. (**D**’,**E**’) Staining of F-actin (green) and N-cadherin (red) in the hair cell nuclei (blue) of the P5 control and P5 T3 group (White arrow showed E-cadherin expressed on pillar cells membrane). The scales in panel (**A**,**D**) represent 10 μm.

E (epithelial)-cadherin and N (neural)-cadherin refer to traditional cadherin molecules, which have been extensively investigated in the inner ear. In addition, changes in these two molecules were detected in the OC. As revealed from the results, although E-cadherin was expressed abundantly on the cell membrane of the IPCs and OPCs, it remained on the cell membrane of the IPCs and OPCs at the later stage (Figure 4A–C). Meanwhile, N-cadherin in the PCs was expressed at a low level in the cytoplasm (Figure 4A’–C’).

To determine whether other cell junction molecules are involved in the early opening of the TC, E-cadherin and N-cadherin were detected in the cochleae of mice treated with T3. As shown in Figure 4D,E, E-cadherin was expressed on the membrane of the PCs in the T3 group and the control. Furthermore, N-cadherin on the PC membrane showed no change in the cochleae of mice treated with T3 (Figure 4D’,E’).

### 2.5. T3 influences the Growth of PCs via Acetylated α-Tubulin

**Figure 5:**
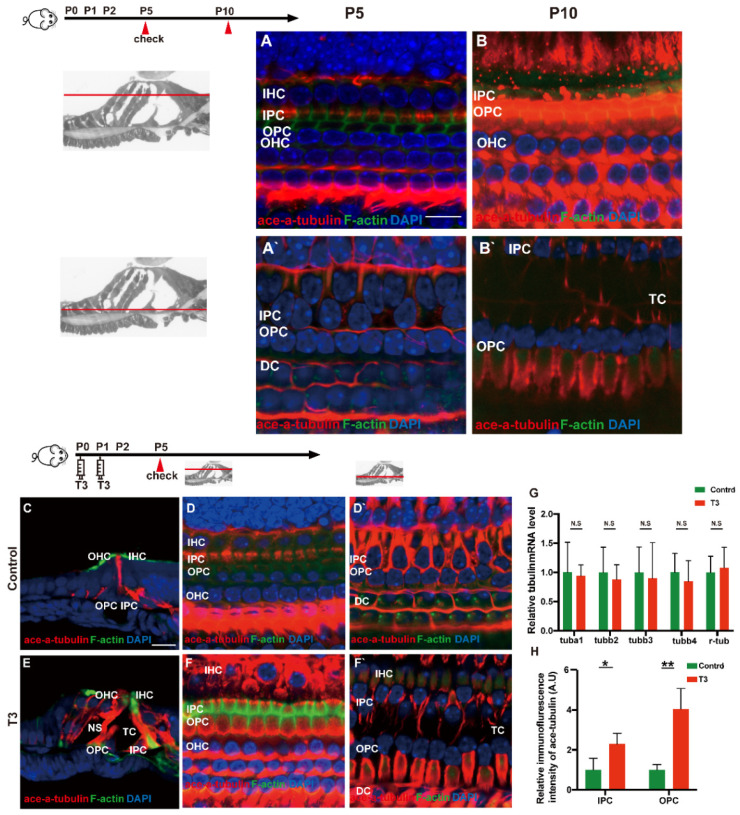
T3 influences the growth of PCs via acetylated α-tubulin. (**A**,**B**) Staining of F-actin (green) and acetylated α-tubulin (red) in hair cells and (**A**’,**B**’) showing those at the layer of PC nuclei (blue) in the P5 and P10 group, respectively. Nuclei were counterstained with DAPI (blue). (**D**,**F**) Staining of F-actin (green) and acetylated α-tubulin (red) in hair cell nuclei (blue) in the P5 control and P5 T3 group, respectively. (**D**’,**F**’) F-actin (green) and acetylated α-tubulin (red) in PC nuclei (blue) in the P5 control and P5 T3 group, respectively. (**C**,**E**) Staining of F-actin (green) and acetylated α-tubulin (red) in sections of the P5 control and P5 T3 group. (**G**) Relative mRNA level of tubule markers in the cochlear basilar membrane in the control and T3 groups (*n =* 6 in each group). (**H**) Quantification of acetylated α-tubulin fluorescence in IPCs and OPCs at the level of the hair cell nuclei (*n =* 3 in each group, * *p* < 0.05, ** *p* < 0.01). The scales in panel (**A**) and (**C**) represent 10 μm and 20 μm, respectively.

Acetylated tubulin was expressed at different levels during OC development and essentially impacted the maturation of the supporting cell structure. At the early stage of cochlear development (P5 and P10), acetylated α-tubulin was detected in the IPCs and OPCs, Deiters’ cells and cochlear nerve fibers (Figure 5A,B). Compared with the OC of mice at P5, the acetylated α-tubulin level of P10 cochleae was elevated in the whole cell body of the IPCs and OPCs.

According to the result of immunofluorescence staining, the expression of acetylated α-tubulin in the IPCs, OPCs and Deiters’ cells was up-regulated in the T3 group at P5 (Figure 5D,F). The expression of acetylated α-tubulin in the top of the IPCs and OPCs (at the layer of hair cells’ nucleus) was significantly up-regulated by 1.291 ± 0.4587 and 305.1 ± 60.24%, respectively (Figure 5H; *p* = 0.0481, 0.0072). Besides considerable immunostaining of nerve fibers, acetylated α-tubulin (red), co-localized with phalloidin (green), was also detected at the bottom of the IPCs and OPCs (at the layer of the inner and outer pillar cells’ nuclei) (Figure 5E). The results of immunofluorescence agreed with the results of transmission electron microscopy, i.e., the expression of acetylated α-tubulin in the IPCs was more than that in the OPCs in the controls at P5, while the expression of acetylated α-tubulin in the IPCs and OPCs was greater than that in the controls at the early stage of T3 induction. However, no significant difference was reported in the mRNA levels of the respective tubulin subtype in the basilar membrane (Figure 5G).

**Figure 6:**
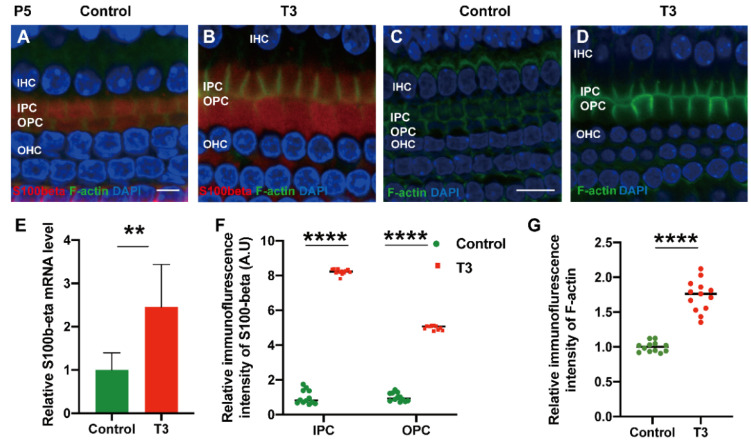
S100β plays in the growth of PCs via acetylated α-tubulin. (**A**,**B**) Immunofluorescence staining of S100β (red) and microfilaments (F-actin, green) in the cochlear basilar membrane of P5 mice in the control group and the T3-treated group, respectively. (**C**,**D**) Microfilament expression (F-actin, green) of P5 in the control group and the T3-treated group, respectively. (**E**) S100β mRNA levels of basilar membrane in the control and T3 group (*n =* 6 in each group). (**F**,**G**) Relative fluorescence intensity of S100β and F-actin in each group at P5 (*n =* 3 in each group). (** *p* < 0.01, **** *p* < 0.0001). Nuclei were counterstained with DAPI (blue). The scales in panel (**A**) and (**C**) represent 5 μm and 10 μm, respectively.

To explore the mechanism via which T3 may affect the mature expression level of tubulin in PCs, molecules with high expression levels in early-stage PCs were selected for the measurement of the mRNA level. S100 calcium-binding protein beta (S100β) was highly expressed in the early stage of cochlear development and may regulate microtubule assembly in vivo. The mRNA levels of S100β in the cochlear basilar membrane of mice at P5 after T3 induction were significantly up-regulated by 145.7 ± 46.76% (Figure 6E, *p* = 0.0109, *n =* 6). Furthermore, the expression of S100β of the T3-treated group was significantly higher, by 401.2 ± 7.664% in the IPCs and 721.8 ± 12.50% in the OPCs, compared to the controls (Figure 6A,D,F, both *p* < 0.0001). The expression of F-actin in the PCs of the T3-treated group was significantly higher by 73.33 ± 6.823% compared to the controls (Figure 6G, *p* < 0.0001).

## 3. Discussion

The normal development of the inner ear involves numerous pathways and relevant functional molecules, but the role played by thyroxine remains to be explored. Classical cadherin molecules include E-cadherin, N-cadherin and P-cadherin, which participate in the morphogenesis of a number of organs by regulating cell morphology, cell differentiation, cell polarity and cell migration. In particular, cadherin primarily forms the intercellular adhesion between epithelial cells and regulates numerous vital aspects of epithelial biology [13]. P-cadherin, a classical adhesion molecule I (immunoglobulin superfamily of adhesion molecules), has not been studied in the inner ear, and the effect of thyroxine on P-cadherin in the inner ear is worth studying in depth. This study revealed that P-cadherin is completely dissipated from the surface of the adjacent OPCs at P8, but is located in the cytoplasm, which may serve as a specific intracellular segment. Moreover, P-cadherin refers to a member of the classic cadherin family and comprises an extracellular segment, a transmembrane segment and a highly conserved intracellular segment that is composed of five extracellular domains containing calcium-binding sites [14]. Additionally, our data show that thyroxine significantly down-regulated P-cadherin on the membrane of PCs and up-regulated the P-cadherin in their cytoplasm. Thyroxine accelerates the decrease in P-cadherin in co-cultured Sertoli cells and gonocytes [15]. There are reasons to believe that P-cadherin is likely to be primarily involved in the degradation of adhesion molecules during the opening of inner ear spaces, and thyroxine seems to regulate the opening of the OC by influencing the expression of P-cadherin in PCs. In previous research, the development of the OC has been suggested to be consistent with the process of epithelial–mesenchymal transition (EMT), i.e., at P8, the E-cadherin molecules in the PCs and Deiters’ cells of the cochleae of rats are reduced [16]. However, our results show that E-cadherin remained on the membrane of the IPCs and OPCs after the OC opening, wrapping around the surface of the supporting cells. In addition, N-cadherin seemed to be expressed at low levels in the cytoplasm instead of in the membrane of PCs. These two types of cadherin are likely to continuously maintain internal and external lymph isolation, assist in the transport and assembly of membrane channels, participate in the connection of the epidermal plate and close the connection of the tip of hair cells [17,18]. Defourny’s group found that the cleavage of E-cadherin might be the reason for opening of the OC [11], and may be responsible for the different levels we observed in the different groups.

As previously reported, the expression of acetylated microtubules was up-regulated significantly during the formation of the OC (P5–P9) [12]. A study on hypothyroid mice showed that low thyroid levels reduced the formation and acetylation of microtubules [6]. Meanwhile, we also found that thyroxine is likely to be capable of increasing the content of acetylated tubulin and F-actin in PCs, as well as making the PCs longer and closer to the mature state. The development of actin filaments and acetylated microtubules increased PC stiffness [12,19]. In the existing studies, many abnormal PCs exhibit pathological phenomena of microtubule formation disorders, e.g., in Gap junction beta-2 protein, (Gjb2) knockout mice and fibroblast growth factor receptor 3 (Fgfr3) knockout mouse models [20,21]. Thus, we consider that thyroxine is involved in the formation of the cytoskeleton of PCs during the early opening of the OC. In the development of PCs, the non-centrosome microtubules gradually occupy most of the space of the cell body, which supports the morphology of PCs and maintains the opening of the OC [22]. The effects of S100α1 and S100β on microtubule formation may be a destructive function, namely the decomposition process of Ca^2+^-dependent microtubules [23]. However, S100β specifically interacts with the microtubules of the centrosome [24]. As indicated by the results, an increase in S100 protein might significantly impact the effect of thyroxine on the development of PCs. This process may involve changes to the tubules in the cytoplasm, which should be further verified experimentally. Thyroxine affects the morphological maturity of the normal OC by promoting the maturation and stage development of the above-mentioned cell structure molecules. Thyroxine affects the development of the nervous system [25], which is also critical to the development of the inner ear. Early studies reported that the location and function of microtubules could be impacted by F-actin, and the microfilaments could be raised by the intracellular segments of adhesion molecules [26,27]. Thyroxine affects formation of the cytoskeleton, nerve regeneration and degradation of adhesion molecules in the testis [28,29], which indicates that thyroxine plays a similar role in the maturation of the OC. As revealed from the results of this study, the thyroxine-induced early opening of the OC involves two processes, i.e., the separation of PCs and the elongation of PCs. In other words, adhesion molecules could fix the cytoskeleton system, including actin on the cell membrane at the interface between the cell and extracellular matrix [30]. Microtubules and microfilaments (F-actin) interact with cadherin molecules on the cell membrane surface through catenin family molecules [31]. Thus, in the PCs, microtubules (acetylated α-tubulin) interact with adhesion molecules (P-cadherin) on the cell membrane via microfilaments, and although the initiation force of the process remains unclear, it is certain that this connection determines whether the OC opens or not. The results also indicate that there is a relationship between the levels of thyroxine in the inner ear and the opening of the OC.

As stated above, microtubules and microfilaments (F-actin) are correlated with cadherin molecules on the cell membrane surface through catenin family molecules [31]. Early studies reported that the location and function of microtubules could be impacted by F-actin, and the microfilaments could be raised by the intracellular segments of adhesion molecules [26]. In other words, adhesion could fix the cytoskeletal system, including actin on the cell membrane of the interface between the cell and extracellular matrix [30]. Consistent with previous results, our data show that there are considerable cytoskeletal systems (including microtubules and microfilaments) in the supporting cells, especially in PCs. The formation of actin filaments and acetylated microtubules increases pillar cell stiffness [12,19]. A study on hypothyroidism in mice showed that a low thyroid hormone level reduced the formation and acetylation of microtubules [6]. Histological studies revealed that the signal transduction mediated by E-cadherin and N-cadherin indirectly stabilize the negative end of microtubules [27]. Thus, in the PCs, microtubules (acetylated α-tubulin) interacted with adhesion molecules (P-cadherin) on the cell membrane via microfilaments. Although the starting force of the process remains unclear, it is certain that this connection determines whether the OC opens or not.

## 4. Materials and Methods

### 4.1. Animals and Models

In this study, wild-type common inbred strain 57, black 6 mice, from the Jackson Laboratory (C57BL/6J), mice were examined on postnatal day (P)2, P5, P7, P9, etc. T3-induced mice were generated through a single subcutaneous injection of 2.0 µg T3 (Sigma-Aldrich, St Louis, MO, USA) diluted in saline to a volume of 20 µL, or the equivalent volume of saline for the controls, at P0 and P1. All experimental procedures were performed in compliance with the policies of the Committee on Animal Research of Tongji Medical College, Huazhong University of Science and Technology.

### 4.2. Immunofluorescent Labelling and Confocal Image Analysis

Cochleae were dissected from the temporal bones and then fixed in 4% paraformaldehyde at 4 °C for 4 h. For flattened cochlear preparation, the cochleae (*n =* 3–4 in each group), after decalcification, were dissected under a stereomicroscope, and two portions of the basilar membrane from the apical and the middle turn were used for PC staining. To prepare cochlear sections, the cochleae were decalcified with disodium ethylenediaminetetraacetic acid (EDTA) and then dehydrated sequentially in a graded sucrose gradient and embedded in OCT. The blocks were then cryosectioned at a thickness of 10 μm. The sections and flattened cochleae were blocked with phosphate-buffered saline (PBS), supplemented with 0.1% bovine serum albumin (BSA) and 1% Triton X-100, and then incubated overnight with the following primary antibodies at 4 °C: P-cadherin, 1:200 (R&D Systems, Minneapolis, MN, USA); acetylated alpha-tubulin, 1:400 (Abcam, Cambridge, MA, USA); N-cadherin, 1:200 (GeneTex, Irvine, CA, USA); E-cadherin; 1:200 (Abcam); and S100β, 1:200 (Proteintech, Rosemont, IL, USA). Next, the primary antibodies were stained using either Alexa Fluor 488- or 647- (1:200, Antgene Biotechnology Company Ltd., Wuhan, PR China) conjugated secondary antibodies. Finally, DAPI (1:200, Biyuntian, PR China) and Phalloidin (0.05 mg/mL, Sigma-Aldrich) were applied to all samples at an ambient temperature for 10 min and 45 min, respectively. Images were captured using a laser scanning confocal microscope (Nikon, Tokyo, Japan) at ×60 magnification under identical conditions. Furthermore, ImageJ (NIH, Bethesda, MD, USA) was used to analyze the average fluorescence intensity from three to four samples.

Data on the immunolabeling of P-cadherin were collected from the original images at the level of hair cell nuclei under identical conditions (e.g., the OPC membrane and PC cytoplasm). For acetylated alpha-tubulin expression in the OC, the size analysis range was used in the cochleae, and this range mainly included the OPCs and IPCs at the level of hair cell nuclei and supporting cell nuclei. For S100β and F-actin expressions in the OC, the same size analysis range was adopted as in the cochleae. Relative fluorescence was quantified by normalizing the ratio of average fluorescence in the induced group to that in the controls. All the data were compared using Student’s *t*-test.

### 4.3. Resin Sections and Transmission Electron Microscopy

The mice used in this study were sacrificed 5 days after T3 injection. The cochleae (*n =* 3 in each group) were isolated and then fixed immediately in 0.1 M phosphate-buffered saline (PBS, pH 7.4), supplemented with 2% paraformaldehyde and 2.5% glutaraldehyde, for 1 h at room temperature and for one night at 4 °C. The inner ear was decalcified for 48–72 h in 10% disodium EDTA (pH 7.2) and then post-fixed for 1 h in 1% osmium tetroxide. The resulting samples were embedded in resin based on an increasing graded ethanol series. The cochleae were then sectioned at 1.5 μm thickness and then stained with Toluidine blue (89640-5G; Sigma-Aldrich). To perform the electron microscopic examination, ultrathin sections were stained with uranyl acetate and lead citrate, and images were captured under an electron microscope (Field Electron and Ion Company Tecnai G2 20 TWIN; Thermo Fisher Scientific, Waltham, MA, USA). To determine the height, length and width of the OC, 10 OCs in the same size region of a section were selected (three to four sections per mouse, three mice per group). All the relevant data were compared by performing Student’s *t*-tests.

### 4.4. RNA Preparation and Quantitative RT-PCR

The membranous labyrinth of the cochlea from 5-day old mice (*n =* 6 for each group) of the experimental groups and the controls was dissected carefully on ice. RNA was extracted using a Total RNA Kit I (Tiangen Biotech Co. Ltd., Beijing, China) by standard procedures. The cDNA was reverse transcribed using a PrimeScript real time (RT) reagent kit with a genomic DNA eraser (TaKaRa Bio Inc., Shiga, Japan). Primer pairs for cadherins and tubulins were as follows: glyceraldehyde 3-phosphate dehydrogenase (Gapdh) forward 5′-GAAGGTCGGTGTGAACGGAT-3′; Gapdh reverse 5′-CTCGCTCCTGGAGATGGTG-3′; P-cadherin forward 5′-AGTGTTCTGGAGGGAGTAATGC-3′; P-cadherin reverse 5′-CCACCACCCCATTGTAAGTG-3′; Tuba1a forward 5′-GTCACAAGGTGCTGCTTCCA-3′; Tuba1a reverse 5′-GAAATGGGCAGCTTGGGTCT-3′; Tubb2a forward 5′-AGGCGGAGAGCAACATGAAT-3′; Tubb2a reverse 5′-ATGCTGGAGGACAACAGAAGTT-3′; Tubb3 forward 5′-AGGCGGAGAGCAACATGAAT-3′; Tubb3 reverse 5′-ATGCTGGAGGACAACAGAAGTT-3′; Tubb4a forward 5′-CGTATGTGACATCCCACCCC-3′; Tubb4a reverse 5′-TTCGAACTCGCCCTCTTCAG-3′; Tubg1 forward 5′-GATGGCCAACCACACCAGTA-3′; Tubg1 reverse 5′-AACCGGTAAGGCAGATGAGG-3′; Actin-β forward 5′-CTTCGCGGGCGACGAT-3′; Actin-β reverse 5′-GACCCATTCCCACCATCACA-3′; S100β forward 5′-TGGTTGCCCTCATTGATGTCT-3′; and S100β reverse 5′-CCCATCCCCATCTTCGTCC-3′. Real-time PCR was performed by employing SYBR Green PCR Technology in a Roche LightCycler^®^ 480 instrument under the following conditions: 95 °C for 5 min; 40 cycles of 95 °C for 20 s; 56 °C for 20 s; and 72 °C for 40 s. Melting curve analysis was conducted using the LightCycler^®^ 480 system to assess the specificity and the integrity of the PCR products. The relative mRNA expression of the experimental groups and the controls was determined by the 2^−ΔΔCt^ method.

### 4.5. Data Analysis

All data are expressed as means ± SD and plotted using GraphPad Prism (Version 8.4.0, GraphPad Software Inc., La Jolla, CA, USA). The *t*-tests were performed with SPSS software (Version 19, IBM SPSS Statistics, IBM Corp., Armonk, NY, USA), and *p* < 0.05 was considered to indicate statistical significance.

## 5. Conclusions

**Figure 7:**
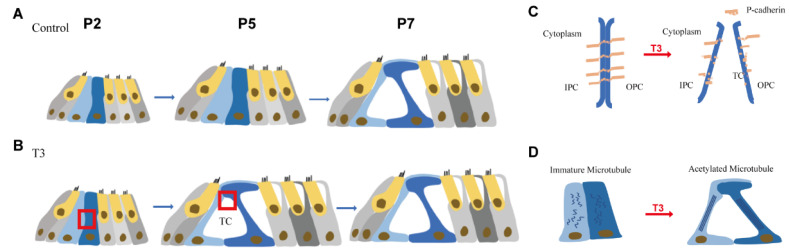
Pattern of T3 regulates the opening of the organ of Corti through modulating P-cadherin and acetylated microtubule. (**A**,**B**) Tunnel of Corti of control mice was opened duringP5 to P7, while T3 promote the opening of TC earlier on P5. (**C**,**D**) shows the detail of red frame in (**B**). (**C**) shows that T3 promoted the process of P-cadherin dissipated from the membrane of PCs. (**D**) shows that T3 increased the acetylated microtubule in PCs, as well as making the PCs longer and closer to the mature state.

The mechanism of interaction between thyroxine, P-cadherin and microtubules in the cochlea is not clear. In this study, we found that acetylated tubulin and P-cadherin affect the opening of the OC, and thyroxine is critical to regulating and controlling this process (Figure 7). Changes in the above processes can lead to cochlear dysfunction. Besides self-exogenous regulation, thyroxine is another specific molecule involved in inner ear development which is worthy of future study. However, we used a thyroxine-induced mice model in this study, and further experiments should be performed to examine the mechanism via hypothyroidism and thyroxine signal disorder models. In conclusion, our study may lead to new treatments for hearing problems attributed to inner ear developmental defects.

## Data Availability

Not applicable.
